# Electroacupuncture at Guanyuan (CV 4), Zusanli (ST 36) and Baihui (DU 20) regulate the aging-related changes in gene expression profile of the hippocampus in sub-acutely aging rats

**DOI:** 10.1371/journal.pone.0191623

**Published:** 2018-01-19

**Authors:** Jianmin Liu, Jing Liu, Guang’an Wang, Guangya Liu, Huanjiao Zhou, Yun Fan, Fengxia Liang, Hua Wang

**Affiliations:** 1 Acupuncture-moxibustion and Orthopedic College, Hubei University of Chinese Medicine, Wuhan, China; 2 Hubei Provincial Collaborative Innovation Center of Preventive Treatment by Acupuncture & Moxibustion, Wuhan, China; 3 The Third Clinical Medicine College, Henan University of Chinese Medicine, Zhengzhou, China; Universidade de Sao Paulo, BRAZIL

## Abstract

To investigate the molecular mechanisms of sub-acutely aging and demonstrate the effect of electroacupuncture (EA) at the Guanyuan (CV 4), Zusanli (ST 36) and Baihui (DU 20) acupoint on the sub-acutely aging brain, cDNA microarrays and bioinformatics analyses were carried out. Thirty Sprague-Dawley (SD) male rats were selected and randomly divided into three groups: the control group (C), the sub-acutely aging model group (M) and the electroacupuncture group (M+EA). Sub-acutely aging model rats were obtained by D-galactose s.c. injection continuously for 40 days. Total RNA was extracted from the hippocampus area of brains in three groups for cDNA microarrays. The data of different groups were compared and analyzed by differential expression analysis, Gene ontology (GO) term enrichment, Kyoto Encyclopedia of Genes Genomes (KEGG) pathway enrichment and quantitative real-time PCR. According to the results, 4052 DE genes were identified in our study. Among them, there were 3079 differentially expressed (DE) genes between group M and group C, and these genes are associated with the aging of rats. Moreover, 983 genes were expressed differently in group M+EA compared with group M, revealing that points stimuli could regulate gene expression in brain with aging. Gene ontology (GO) term enrichment and KEGG enrichment were performed to further classify the differential expression genes. Important GO terms and KEGG pathways connected with sub-acutely aging EA effects were identified. At last, 3 significant differentially expressed genes were selected for real-time quantitative PCR to clarify the cDNA microarray results. In conclusion, the cDNA microarray data first compared and analyzed the differences of gene expression profile in the hippocampus of rats in different groups, which contribute to our knowledge on the molecular mechanisms of EA towards sub-acutely aging.

## Introduction

In recent years, the number of older persons is increasing rapidly. Under this circumstance, the problem of aging is drawing more and more social attention. Aging is accompanied by cognitive decline in a major segment of the population and is the primary risk factor for Alzheimer's disease and other prevalent neurodegenerative disorders. As people become older, the age-related changes will occur, such as changes in the functions and composition of the human body [1, 2]. Coupled with physiologic changes, there are also medical conditions that rise more commonly with advancing age. These changes and conditions increase an older adult’s vulnerability to and injuries from abuse or neglect [3]. According to formal research, aging is associated with deterioration of the immune system (immunosenescence), an increased susceptibility to infection, autoimmune disease and cancer and reduced responsiveness to vaccination [4, 5]. A key feature of the aged human immune system is the accumulation of highly differentiated CD8(+)CD28(-) T cells, a phenomenon that negatively influences immune function in the elderly. As a result, the mechanisms that regulate survival or death of CD8(+)CD28(-) T cells has become the focus of many researchers [6]. To combat immunosenescence, a lot of strategies are emerging, focusing on cellular and genetic therapies, which include bone marrow transplantation and genetic reprogramming [7]. Except for immunosenescence, brain aging processes are also enormously complex phenomena that can include cognitive decline and increase the risk of Alzheimer’s disease (AD) [8]. There are different kinds of methods to combat age-related brain diseases or delay brain aging, including drug therapy and non-drug therapy [9]. For instance, Mannosylated liposomal flavonoid is useful in combating age-ralated ischemia-reperfusion induced oxidative damage in rat brain [10]. Moreover, Caloric restriction (CR) and transgenic over expression of endogenous antioxidants therapy were proven to delay or inhibit a number of age-associated pathologic and biologic changes in the brain, thus to have life-extending function [11, 12].

Acupuncture is a traditional therapy applied for thousands of years, and it was also a powerful non-drug therapy which is used extensively in Oriental Medicine and has emerged as an important modality of complementary and alternative therapy to Western Medicine [13]. In the theory of traditional Chinese medicine, it is proposed that acupuncture can strengthen the human body to resist diseases by puncturing needles at certain points [14]. It has also been proved that acupuncture theory could apply to rats and that the results of acupuncture experiments based on rats agree with the results based on human. Until now, rats’ models have been applied to the acupuncture research of various kinds of diseases, such as hypertension, Alzheimer disease, diabetes, aging, and have achieved great success [15]. For example, previous research demonstrated that acupuncture at “Taichong” (LR3) has immediate effect on patients with hypertension of 1 or 2 degree, and the lowering extent is positively related with the blood pressure before acupuncture [16]. Then other research groups proved that moderate-stimulation of “Taichong” (LR 3) can lower blood pressure and plasma EA-1 level in hypertension rats. So the spontaneously hypertensive rats (SHR) model was used by many researchers to seek a better acupuncture parameter for clinical treatment of hypertension [17, 18]. Double reinforcing-one unblocking acupuncture was proved to have a significantly effect on senile immunologic function [19]. Other researches demonstrated that double reinforcing-one unblocking acupuncture could also improve the spatial learning and memory in aging model rats [20, 21].

So far, the function of acupuncture in regulating the immune system has been revealed in many researches. For instance, acupuncture can enhance anticancer and antistress immune function and exert antiinflammation effects. The acupuncture point ST36 (Zusanli) is an important acupoint which is widely applied in immune-related diseases. M. Zhu et al. reported that EA preconditioning at ST36 obviously ameliorated CLP-induced intestinal injury and high permeability and reduced the mortality of CLP-induced sepsis rats through increasing the concentration of sIgA and the percentage of CD3+, γ/δ, and CD4+ T cells and the ratio of CD4+/CD8+ T cells [22]. Another research group discovered a novel cholinergic anti-inflammatory pathway activated by acupuncture as well as a chemokine-mediated proliferation of opioid-containing macrophages in inflamed tissues in response to acupuncture [23]. Acupuncture was proved to play a role in delaying brain aging and treating age-related brain diseases. “*YiQi-TiaoXue*, *FuBenPeiYuan*” acupuncture method has been proved to improve cognition ability of dementia rat [24]. Moreover, acupuncture can induce different cell proliferation in different brain regions of SAMP8, which brings forth the need to explore further for the mechanism of cognitive deficits and acupuncture intervention in this field [25].

Based on a long clinic experience and studying of the traditional Chinese medicine theory, the electroacupuncture treatment which insert points at Guanyuan (CV 4), Zusanli (ST 36) and Baihui (DU20) with needle, also named “Shuanggu Yitong” (double-reinforcing and one-unblocking) acupuncture therapy has been found the special effect to regulate the immune system for rats [26–28]. Some mechanisms of how the “Shuanggu Yitong” electroacupuncture therapy regulates the immunosenescence have been studied in our previous research. It turns out that EA at Guanyuan (CV 4), Zusanli (ST 36) and Baihui (DU 20) could delay aging by regulating T cells in proliferation, secretion of IL-2 and its receptor and improve the expression of CD8^+^, CD28^+^ [22, 23]. Further study reveals that EA at Guanyuan (CV 4), Zusanli (ST 36) and Baihui (DU 20) could decrease the content of IL-1β and IL-6 of serum [28]. Based on the previous researches and in order to gain a better understanding of aging-related changes and how “Shuanggu Yitong” EA affects the brains of sub-acutely aging rats, DNA microarray analysis was explored. The microarray data demonstrate differences between control group (C), the electroacupuncture group (M+EA) and the sub-acutely aging model group (M). The differences existed in gene expression level, GO categories and pathway categories, which contribute to our knowledge on the effect of EA to the brain of sub-acutely aging rats in the molecular level. Important genes related with sub-acutely aging and EA effects were selected for further study.

## Materials and methods

### Animal treatment

Thirty SD male rats in 3-month-old were obtained from the Animal Experimental Center of Hebei Medical University, China [Experimental animal production license number: SCXK(Hebei)2008-1-003]. All the rats were raised in the individually ventilated cages with the temperature between 20°C to 25°C.The humidity was between 45% and 55%. Light was provided from 8 am to 8 pm to simulate the circadian rhythms while food and water was offered sufficiently. All animal treatments were approved by the Animal Ethics Committee of the Hubei University of Chinese Medicine, No. [2016] IEC (010).

After 1 week of adaptation, the rats were randomly divided into three groups as following: the control group (C), the sub-acutely aging model group (M) and the electroacupuncture group (M+EA), 10 rats for each group. Rats in group C were raised normally without any treatments. While the sub-acutely aging model rats were obtained by D-galactose s.c. injection continuously for 40 days since the second week. The concentration of D-galactose s.c. was 350mg.kg-1.d-1, one time for each day [29]. Rats in group M were raised without any treatments after modeling. While the Rats of M+EA group started EA treatment at Guanyuan (CV 4), Zusanli (ST 36) and Baihui (DU 20) once a day after modeling for 27 days (the procedures are shown as following). The process of the experiment was shown in S1 Fig. The body temperature, tongue temperature and the weight of rats in different groups were measured every three days during the model making and electroacupunture procedures to make sure that the physiological indicators of rats are normal. All efforts were made to minimize suffering.

### Electroacupuncture (EA) treatment

Sterile acupuncture needles [size:φ0.30*25 mm (diameter:0.30 millimeter, length: 25 millimeter); made by Suzhou Acupuncture & Moxibustion Appliance Co., Lid, Suzhou, P.R. China] and the pulse generator (6805-II, Shanghai Taicheng technology development co., LTD) were used during the EA treatment. Needles were inserted perpendicularly into the muscle layer at Guanyuan (CV 4), Zusanli (ST 36) and inserted horizontally into subgaleal tissue at the point of Baihui (DU 20) to a depth of 2 mm. The Guanyuan (CV 4) acupoint was connected to the negative charge of the pulse generator and the Zusanli (the bilateral Zusanli were used alternately in the EA treatment) was connected to the positive charge (continuous wave: 2 Hz, 1 mA, lasted 15 min). Baihui (DU 20) was stimulated with needles at the same time: After the needle has reached its desired location, to twirl and rotate the needle backward and forward continuously with the frequency 2–3 times per second for 30 seconds. The same manipulation was done after every 5 minutes for three times until withdrawing the needle. The EA treatment was performed every day except Sunday and lasted for 27 days. This electroacupuncture (EA) treatment protocal has been deposited in the protocols.io. The digital object identifier (DOI) link is: http://dx.doi.org/10.17504/protocols.io.ky8cxzw.

### Tissue sampling

At the end of the EA treatment, rats from three groups were fasted overnight. Then all the rats were killed by dislocation of cervical vertebra and the brains were quickly excised after intraperitoneal anesthesia using pentobarbital natrium. The collected samples were washed by the cold normal saline. Subsequently, the hippocampus area was divided, frozen in the liquid nitrogen and then kept under -80°C for RNA extraction. The hippocampus area of ten rats in each group makes up one sample, and then the three samples from group C, group M and group M+EA were prepared for the RNA extraction.

### RNA preparation and quantitative real-time PCR (q-PCR)

RNAiso Plus (TaKaRa Biotech. Co., Dalian, China) was used to extract the total RNA according to the manufacturer’s protocol. All RNA samples were treated with DNase I (TaKaRa Biotech. Co., Dalian, China) and frozen at −80°C before DNA microarray experiment. RNA quantity and purity was assessed using NanoDrop ND-1000 (Pass criteria for absorbance ratios are established at A260/A280 ≥ 1.8 and A260/A230 ≥ 1.6). RIN values are ascertained using Agilent RNA 6000 Nano assay to determine RNA integrity (Pass criteria for RIN value is established at ≥ 7 indicating acceptable RNA integrity). gDNA contamination was evaluated by gel electrophoresis.

First-strand cDNA was prepared by All-in-one First strand cDNA Synthesis Kit (Genecopoeia, Guangzhou, China) following the manufacturer’s protocol. Then the BIO-RAD CFX96 q-PCR system (SYBR Green I fluorescent dye detection) was used to perform the qRT-PCR. The mRNA abundance was normalized with the housekeeping gene β-actin, and the relative expression levels were calculated using the 2-ΔΔCt method [30].

### Microarray

Microarray analysis was carried out to investigate gene expression patterns in the hippocampus of rats in different groups. 6-OHDA-targeted transcripts were analyzed with Whole Rat Genome 4 × 44 K microarrays (Gene expression hybridization kit, Agilent) according to the manufacturer’s instruction. Rosetta Resolver SystemR (Rosetta Biosoftware) was used to process data analysis: 1. Rosetta profile error model calculation: the error due to random factors and systematic biases are estimated by Rosetta error model which can capture the predictable behavior of the variance in microarray measurement [31]; 2. Squeeze replicated probes: the repeated probes within one chip are averaged; 3. Normalize intensities: Median scaling performed on data set without flagged and control data; 4. Pearson’s correlation coefficient: statistical analysis calculated on three technical replicates to assess reproducibility; The replicated analysis of the RNA from the same sample can decrease the technical variation and the false positive, making the results more accurately. 5. Merge technical replicate data: Average intensity values calculated on technical replicates; 6. Pairwise ratio calculation: Probe filtering, normalization, pair-wise comparison and error-weighted modeling are performed based on customers’ designated sample groups; 7. Differentially expressed gene lists: Standard selection criteria to identify differentially expressed genes are established at |Fold change| ≥ 1 and P < 0.05. Student’s t-test (two-tailed) was used for data analysis this study. 8. The heat map was obtained using the software HemI [32], while the Venn diagram was obtained using VENNY [33]. We applied KOBAS software to test the statistical enrichment of differential expression genes in KEGG pathways.

## Results

### RNA preparation

Total RNA was extracted from the hippocampus of rats in different groups and treated with DNase I. The absorbance ratios of A260/A280 in group C, M and M+EA were 1.99, 1.99 and 1.88, respectively. While the absorbance ratios of A260/A230 were 1.98, 2.16 and 1.96. RIN values in three groups were all above the pass criteria (8.5, 9.0 and 8.3 respectively) **(Table 1)**. The results indicating that the RNA purity and integrity are suitable for cDNA microarray experiment.

**Table 1:** Quality control of RNA sample in three groups.

Sample	A260/A280	A260/A230	RIN	Conc(ng/ul)
Group C	1.99	1.98	8.5	1042.7
Group M	1.99	2.16	9.0	734.0
Group M+EA	1.88	1.96	8.3	705.8

### Identification of differentially expressed genes between different groups

**Fig 1:**
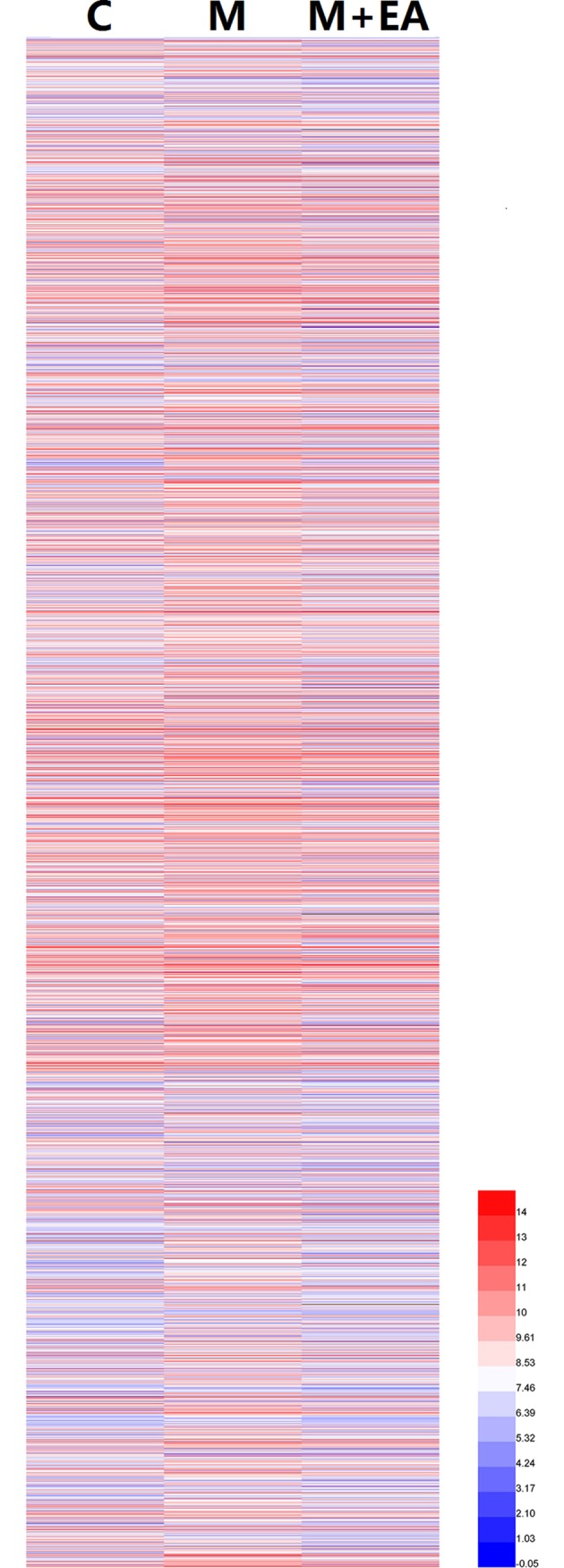
Heat map of differentially expressed genes of three samples.

To demonstrate the molecular mechanisms of the sub-acutely aging and EA effects, the differentially expressed (DE) genes between different groups were identified and analyzed. Firstly, the heat map was made to compare the expression patterns of DE genes in three groups. There are 4052 DE genes identified in our study (**S1 Table**). As can be seen in **Fig 1**, the gene expression pattern of group M was quite different from that of group C, revealing that the sub-acutely aging was associated with the expression changing of a large sum of genes in the hippocampus. Meanwhile, the expression level of DE genes in group M+EA was different from that in group M, which suggested that EA has changed the gene expression pattern of brain in sub-acutely aging rats.

**Fig 2:**
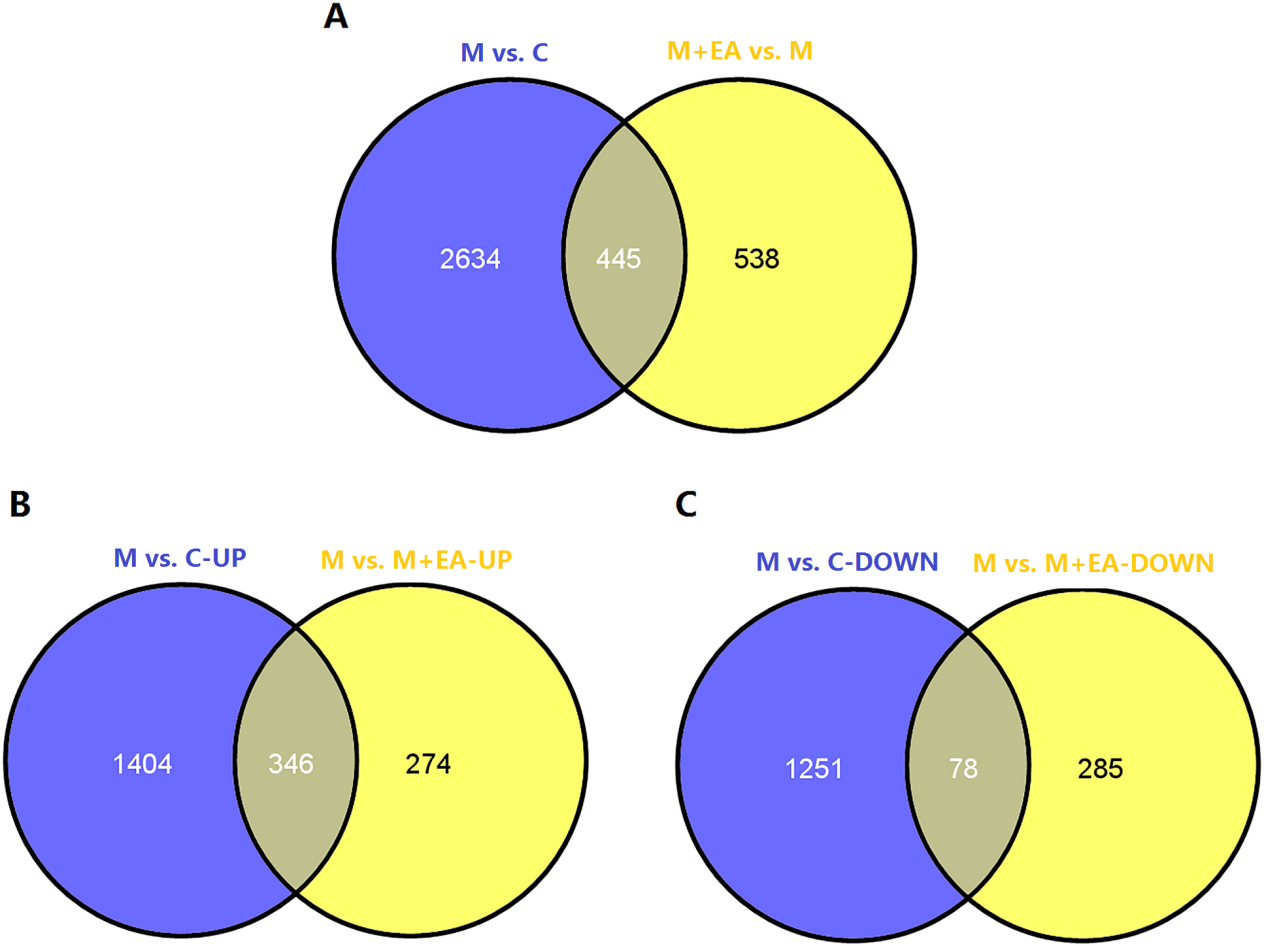
Comparisons of the number and overlapping relationships of DE genes between different samples. A. Purple circle represents number of DE genes between group M and group C; yellow circle stand for number of DE genes between group M+EA and group M; B. Purple circle represents number of up-regulated DE genes between group M and group C; yellow circle stand for number of up-regulated DE genes between group M and group M+EA; C. Purple circle represents number of down-regulated DE genes between group M and group C; yellow circle stand for number of down-regulated DE genes between group M and group M+EA. The overlapping region represents shared DE genes of two comparable groups.

According to the results, there were 3079 DE genes between group M and group C, among them 1750 genes were up-regulated and 1329 were down-regulated. These genes are related with the aging of brain in rats. When comparing group M+EA and group M, 983 DE genes were identified. The number of up-regulated genes was 620 while the number of down-regulated genes was 363, as shown in **Table 2**. Further analysis was made to compare the number and overlapping relationships of DE genes between different groups. According to **Fig 2A**, the comparing groups M vs. C and M+EA vs M shared 445 DE genes. 2634 DE genes were peculiar to comparing group M vs. C and 538 DE genes were peculiar to M+EA vs M. Then the shared 445 DE genes were analyzed to figure out important genes related with sub-acutely aging and EA effects. By comparing the up-regulated genes between M vs. C and M vs. M+EA, we identified 348 overlapping DE genes, which mean EA can reverse the down-regulate of these genes in the sub-acutely aging group. Besides, 1404 DE genes were peculiar to M vs. C comparing group and 274 were peculiar to M vs. M+EA **(Fig 2B)**. Likewise, by comparing the down-regulated genes of M vs. C and M vs. M+EA, 78 shared DE genes were identified. 1251 DE genes were peculiar to M vs. C down-regulated genes and 285 were peculiar to comparing group M vs. M+EA **(Fig 2C)**. The result reveals that EA treatment can up-regulate the expression of 78 genes which were down-regulated in group M. Common elements in “M vs C-UP” and “M vs M+EA-UP” and in “M vs C-DOWN” and “M vs M+EA-DOWN” were shown in **S2 Table**.

**Table 2:** The number of DE genes between different samples.

NO	Comparison	Up-regulated	Down-regulated
1	C vs. M+EA	1164	945
2	M vs. M+EA	363	620
3	M vs. C	1750	1329

### Functional distribution of differentially expressed genes

The identified differentially expressed genes were further analyzed by Gene ontology (GO) term enrichment and Kyoto Encyclopedia of Genes Genomes (KEGG) pathway enrichment. Comparing groups M vs C and M+EA vs M were chosen for GO and KEGG analysis. According to the GO categories, the identified DE genes were categorized into three major functional groups: cellular component, molecular function, and biological process. In comparing group M vs C, the abundant genes were categorized into 20 major functional groups based on the GO categories, and protein import into nucleus translocation, trans golgi network transport vesicle and kinase activoter acvitity are the top three functional categories, as can been seen in **Table 3**. Likewise, in comparing group M+EA vs M, the abundant DE genes were categorized into 20 major functional groups, and functional mategories are positive regulation of transport, voltage gated potassium channel complex and vitamin binding are the most abundant (**Table 4**).

**Table 3:** The most enriched GO terms between group M and group C.

Biological Process	Cellular Component	Molecular Function
PROTEIN_IMPORT_INTO_NUCLEUS_TRANSLOCATION	TRANS_GOLGI_NETWORK_TRANSPORT_VESICLE	KINASE_ACTIVATOR_ACTIVITY
INTERACTION_WITH_HOST	TRANSPORT_VESICLE	SERINE_TYPE_ENDOPEPTIDASE_ACTIVITY
HEMOSTASIS	ENDOCYTIC_VESICLE	RAS_GUANYL_NUCLEOTIDE_EXCHANGE_FACTOR_ACTIVITY
ACUTE_INFLAMMATORY_RESPONSE	NUCLEAR_CHROMATIN	VITAMIN_BINDING
REGULATION_OF_RAS_PROTEIN_SIGNAL_TRANSDUCTION	BASOLATERAL_PLASMA_MEMBRANE	SERINE_TYPE_PEPTIDASE_ACTIVITY
CDC42_PROTEIN_SIGNAL_TRANSDUCTION	LAMELLIPODIUM	SERINE_HYDROLASE_ACTIVITY
NLS_BEARING_SUBSTRATE_IMPORT_INTO_NUCLEUS	OUTER_MEMBRANE	GUANYL_NUCLEOTIDE_EXCHANGE_FACTOR_ACTIVITY
SMOOTH_MUSCLE_CONTRACTION_GO_0006939	GOLGI_ASSOCIATED_VESICLE	METABOTROPIC_GLUTAMATEGABA_B_LIKE_RECEPTOR_ACTIVITY
BLOOD_COAGULATION	EARLY_ENDOSOME	CARBON_CARBON_LYASE_ACTIVITY
COAGULATION	SECRETORY_GRANULE	GABA_RECEPTOR_ACTIVITY
WOUND_HEALING	NUCLEAR_MEMBRANE_PART	OXIDOREDUCTASE_ACTIVITY_ACTING_ON_PEROXIDE_AS_ACCEPTOR
PROTEIN_IMPORT_INTO_NUCLEUS	TIGHT_JUNCTION	RHO_GUANYL_NUCLEOTIDE_EXCHANGE_FACTOR_ACTIVITY
REGULATION_OF_SMALL_GTPASE_MEDIATED_SIGNAL_TRANSDUCTION	CONDENSED_CHROMOSOME	INWARD_RECTIFIER_POTASSIUM_CHANNEL_ACTIVITY
NUCLEAR_IMPORT	CENTROSOME	NEUROPEPTIDE_HORMONE_ACTIVITY
REGULATION_OF_BODY_FLUID_LEVELS	APICAL_JUNCTION_COMPLEX	PHOSPHATE_TRANSMEMBRANE_TRANSPORTER_ACTIVITY
POSITIVE_REGULATION_OF_TRANSPORT	APICOLATERAL_PLASMA_MEMBRANE	MRNA_BINDING
NEGATIVE_REGULATION_OF_MULTICELLULAR_ORGANISMAL_PROCESS	PORE_COMPLEX	NEUROPEPTIDE_RECEPTOR_ACTIVITY
NEGATIVE_REGULATION_OF_CELL_CYCLE	CHROMATIN	CHROMATIN_BINDING
FEEDING_BEHAVIOR	CLATHRIN_COATED_VESICLE	NEUROPEPTIDE_BINDING
MITOTIC_SISTER_CHROMATID_SEGREGATION	ORGANELLE_OUTER_MEMBRANE	SMALL_GTPASE_BINDING

**Table 4:** The most enriched GO terms between group M and group C.

Biological Process	Cellular Component	Molecular Function
POSITIVE_REGULATION_OF_TRANSPORT	VOLTAGE_GATED_POTASSIUM_CHANNEL_COMPLEX	VITAMIN_BINDING
INTERACTION_WITH_HOST	TRANS_GOLGI_NETWORK_TRANSPORT_VESICLE	AUXILIARY_TRANSPORT_PROTEIN_ACTIVITY
ACUTE_INFLAMMATORY_RESPONSE	ENDOCYTIC_VESICLE	VOLTAGE_GATED_POTASSIUM_CHANNEL_ACTIVITY
POTASSIUM_ION_TRANSPORT	ENDOSOME	METABOTROPIC_GLUTAMATEGABA_B_LIKE_RECEPTOR_ACTIVITY
CELL_FATE_COMMITMENT	EARLY_ENDOSOME	POTASSIUM_CHANNEL_ACTIVITY
NEGATIVE_REGULATION_OF_MULTICELLULAR_ORGANISMAL_PROCESS	VESICULAR_FRACTION	GUANYL_NUCLEOTIDE_EXCHANGE_FACTOR_ACTIVITY
REGULATION_OF_TRANSFORMING_GROWTH_FACTOR_BETA_RECEPTOR_SIGNALING_PATHWAY	COLLAGEN	INWARD_RECTIFIER_POTASSIUM_CHANNEL_ACTIVITY
DIGESTION	MICROSOME	KINASE_ACTIVATOR_ACTIVITY
FEEDING_BEHAVIOR	CENTROSOME	LOW_DENSITY_LIPOPROTEIN_BINDING
ORGANELLE_LOCALIZATION	GOLGI_ASSOCIATED_VESICLE	NEUROPEPTIDE_HORMONE_ACTIVITY
MONOVALENT_INORGANIC_CATION_TRANSPORT	TRANSPORT_VESICLE	SMAD_BINDING
ESTABLISHMENT_OF_ORGANELLE_LOCALIZATION	TIGHT_JUNCTION	PHOSPHOLIPASE_A2_ACTIVITY
REGULATION_OF_RAS_PROTEIN_SIGNAL_TRANSDUCTION	CELL_SURFACE	POTASSIUM_CHANNEL_REGULATOR_ACTIVITY
CELLULAR_RESPONSE_TO_NUTRIENT_LEVELS	BASOLATERAL_PLASMA_MEMBRANE	SOLUTE_SODIUM_SYMPORTER_ACTIVITY
CELLULAR_RESPONSE_TO_STRESS	MICROTUBULE_ORGANIZING_CENTER	RAS_GTPASE_BINDING
TRIACYLGLYCEROL_METABOLIC_PROCESS	APICAL_JUNCTION_COMPLEX	COPPER_ION_BINDING
COFACTOR_TRANSPORT	APICOLATERAL_PLASMA_MEMBRANE	PEPTIDE_RECEPTOR_ACTIVITY
RESPONSE_TO_EXTRACELLULAR_STIMULUS	CLATHRIN_COATED_VESICLE	LIPID_TRANSPORTER_ACTIVITY
REGULATION_OF_SMALL_GTPASE_MEDIATED_SIGNAL_TRANSDUCTION	EXTRACELLULAR_SPACE	SERINE_TYPE_ENDOPEPTIDASE_ACTIVITY
CDC42_PROTEIN_SIGNAL_TRANSDUCTION	COATED_VESICLE	SODIUM_CHANNEL_ACTIVITY

**Fig 3:**
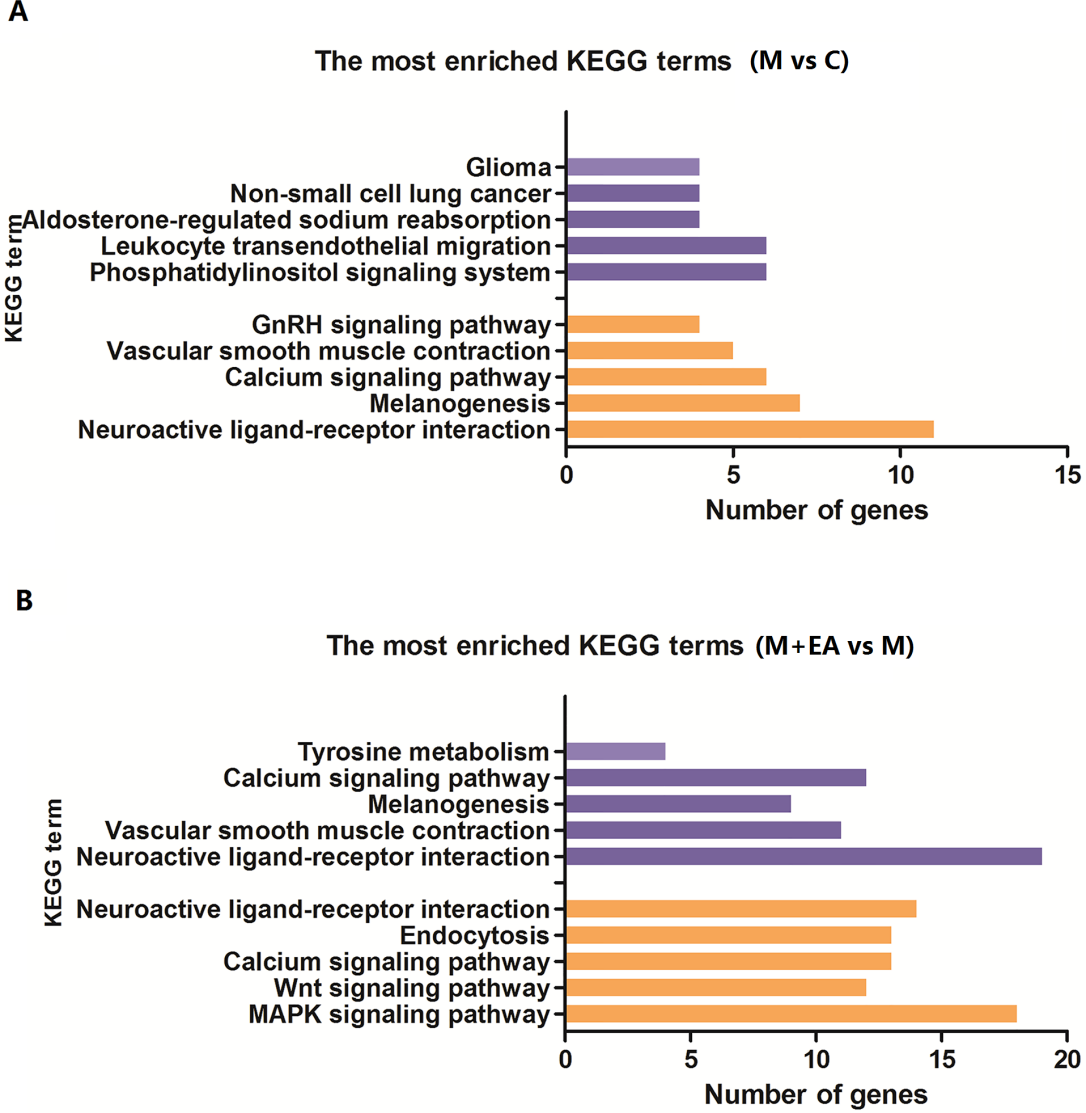
The top ten most enriched KEGG terms in each comparable group. A: Comparable group: group M vs group C; B: Comparable group: group M+EA vs group M. The purple bars represent the up-regulated KEGG pathways; the orange bars represent the down-regulated KEGG pathways.

The KEGG pathway enrichment were then performed to category the DE genes in comparing group M vs C and M+EA vs M. Compared with group C, 5 KEGG pathway were identified up-regulated and 9 were identified down-regulated in group M. The up-regulated terms were as following: rno04080: Neuroactive ligand-receptor interaction, rno04916: Melanogenesis, rno04270: Vascular smooth muscle contraction, rno04020: Calcium signaling pathway and rno04912: GnRH signaling pathway; the down-regulated terms were: rno04070: Phosphatidylinositol signaling system, rno04670: Leukocyte transendothelial migration, rno04960: Aldosterone-regulated sodium reabsorption, rno05223: Non-small cell lung cancer, rno05214: Glioma, rno05200: Pathways in cancer, rno04662: B cell receptor signaling pathway and rno05222: Small cell lung cancer (**Fig 3A**). DE genes involved in these pathways were listed in **Table 5**. In comparing group M+EA vs M, 18 KEGG pathway terms were up-regulated, and the most abundant five terms were rno04070: Phosphatidylinositol signaling system, rno04720: Long-term potentiation, rno04010: MAPK signaling pathway, rno04310: Wnt signaling pathway and rno04020: Calcium signaling pathway. Compared with group M, 5 KEGG pathway terms were down-regulated in group M+EA, including rno04080: Neuroactive ligand-receptor interaction, rno04270: Vascular smooth muscle contraction, rno04916: Melanogenesis, rno04020: Calcium signaling pathway and rno00350: Tyrosine metabolism, as shown in **Fig 3B** and **Table 5**. Notably, while the gene expression in phosphatidylinositol signaling system pathway was down-regulated in group M compared with group C, it was up-regulated after the EA treatment. Besides, while KEGG pathways neuroactive ligand-receptor interaction, vascular smooth muscle contraction, melanogenesis and calcium signaling pathway were up-regulated in the sub-acutely model group, they were all down-regulated after the EA treatment. The results reveal that EA treatment can affect the expression of genes in these KEGG pathways, and reversing the gene expression changes in these pathways can be regarded as one of the mechanisms of EA to against brain sub-acutely aging. Important genes involved in these five pathways will be further studied.

**Table 5:** The most enriched KEGG terms in comparing groups M vs C and M+EA vs M.

Comparing group	Term	Count	%	Genes
M vs C-Up	Neuroactive ligand-receptor interaction	11	6.58	CALCR, HCRTR2, EDNRB, GABRR1, PTH2R, AGTR1A, GLRA2, P2RX2, GPR50, MC3R, TSHR
M vs C-Up	Melanogenesis	7	4.19	DCT, EDNRB, WNT3, PLCB4, ADCY7, GNAS, POMC
M vs C-Up	Vascular smooth muscle contraction	5	2.99	RAMP3, PLCB4, ADCY7, AGTR1A, GNAS
M vs C-Up	Calcium signaling pathway	6	3.59	EDNRB, PLCB4, ADCY7, AGTR1A, P2RX2, GNAS
M vs C-Up	GnRH signaling pathway	4	2.39	PLCB4, GNRH1, ADCY7, GNAS
M vs C-Down	Phosphatidylinositol signaling system	6	0.32	PIK3CD, SYNJ2, INPP5D, ITPKA, PIK3R2, PRKCB
M vs C- Down	Leukocyte transendothelial migration	6	0.32	PTK2B, MMP9, PIK3CD, CXCL12, PIK3R2, PRKCB
M vs C- Down	Aldosterone-regulated sodium reabsorption	4	0.21	PIK3CD, NEDD4L, PIK3R2, PRKCB
M vs C- Down	Non-small cell lung cancer	4	0.21	E2F3, PIK3CD, PIK3R2, PRKCB
M vs C- Down	Glioma	4	0.21	E2F3, PIK3CD, PIK3R2, PRKCB
M vs C- Down	Pathways in cancer	8	0.42	WNT2, WNT10A, E2F3, MMP9, PIK3CD, LAMC2, PIK3R2, PRKCB
M vs C- Down	B cell receptor signaling pathway	4	0.21	PIK3CD, INPP5D, PIK3R2, PRKCB
M vs C- Down	Small cell lung cancer	4	0.21	E2F3, PIK3CD, LAMC2, PIK3R2
M+EA vs M-Up	Phosphatidylinositol signaling system	11	2.10	PIK3CD, PIP5K1C, SYNJ2, DGKZ, PRKCG, INPP5D, PLCB1, ITPKA, PIP4K2C, ITPR1, PRKCB
M+EA vs M-Up	rno04720:Long-term potentiation	9	1.72	GRIA2, CAMK4, GRIA1, PPP1R1A, GRIN1, PRKCG, PLCB1, ITPR1, PRKCB
M+EA vs M-Up	MAPK signaling pathway	18	3.44	MEF2C, NLK, MRAS, CACNB1, MAP4K1, NR4A1, PRKCG, CACNG3, MAPK10, HSPA1B, PRKCB, DUSP5, FOS, BDNF, RASGRF2, DUSP1, DUSP9, DUSP6
M+EA vs M-Up	Wnt signaling pathway	12	2.29	WNT2, TBL1XR1, WNT10A, WNT16, PRICKLE1, CCND2, NLK, PRKCG, MAPK10, WNT9A, PLCB1, PRKCB
M+EA vs M-Up	Calcium signaling pathway	13	2.49	GNA14, GRIN1, PRKCG, ITPKA, ITPR1, PRKCB, ADRB3, CAMK4, PTK2B, PDE1A, PLCB1, ADRA1D, HTR5B
M+EA vs M-Up	Endocytosis	13	2.49	GIT1, PIP5K1C, VPS37B, HSPA1B, SRC, ADRB3, ACAP3, PSD, NEDD4L, AGAP2, DNM1, COL20A1, SH3GL1
M+EA vs M-Up	Glycosphingolipid biosynthesis	4	0.76	ST3GAL5, ST8SIA5, SIAT7E, B4GALNT1
M+EA vs M-Up	Fc gamma R-mediated phagocytosis	8	1.53	WASF1, PIK3CD, PIP5K1C, PRKCG, INPP5D, PRKCE, DNM1, PRKCB
M+EA vs M-Up	Long-term depression	7	1.34	GRIA2, GRIA1, GRIA3, PRKCG, PLCB1, ITPR1, PRKCB
M+EA vs M-Up	Inositol phosphate metabolism	6	1.14	PIK3CD, PIP5K1C, SYNJ2, PLCB1, ITPKA, PIP4K2C
M+EA vs M-Up	Neuroactive ligand-receptor interaction	14	2.6	GRIN1, GRIA3, VIPR1, SSTR4, ADRB3, SSTR2, CHRM4, GRIA2, SSTR1, GRIA1, MAS1, ADRA2C, ADRA1D, HTR5B
M+EA vs M-Up	Axon guidance	9	1.72	EPHB6, EPHA6, SEMA7A, SEMA3E, EFNA3, ROBO2, CXCL12, SLIT1, SLIT3
M+EA vs M-Up	Melanogenesis	7	1.34	WNT2, WNT10A, WNT16, PRKCG, WNT9A, PLCB1, PRKCB
M+EA vs M-Up	Regulation of actin cytoskeleton	11	2.10	GIT1, CHRM4, ARHGEF7, MRAS, BAIAP2, WASF1, PIK3CD, ITGA11, PIP5K1C, ACTN2, PIP4K2C
M+EA vs M-Up	Adherens junction	6	1.14	BAIAP2, NLK, WASF1, LMO7, ACTN2, SRC
M+EA vs M-Up	Tight junction	8	1.53	MRAS, MYH3, MYH2, PRKCG, ACTN2, PRKCE, SRC, PRKCB
M+EA vs M-Up	Circadian rhythm	3	0.57	NPAS2, PER2, PER1
M+EA vs M-Up	Focal adhesion	10	1.91	CCND2, PIK3CD, ITGA11, RELN, LAMC2, PRKCG, ACTN2, MAPK10, SRC, PRKCB
M+EA vs M-Down	Neuroactive ligand-receptor interaction	19	4.13	CALCR, PTH2R, DRD2, GLRA2, NTSR1, GRM1, HCRTR2, EDNRB, GRM4, P2RX6, PRLR, AGTR1A, HTR7, P2RY1, P2RX2, AVPR1A, GPR50, MC3R, TSHR
M+EA vs M-Down	Vascular smooth muscle contraction	11	2.39	RAMP3, PRKCQ, PLCB4, ADCY7, AGTR1A, GNA12, MRVI1, AVPR1A, PRKCH, GNAS, PRKCD
M+EA vs M-Down	Melanogenesis	9	1.96	DCT, EDNRB, WNT3, PLCB4, ADCY7, WNT9B, GNAS, POMC, ASIP
M+EA vs M-Down	Calcium signaling pathway	12	2.61	GNAL, EDNRB, P2RX6, PLCB4, ADCY7, AGTR1A, HTR7, P2RX2, AVPR1A, GNAS, NTSR1, GRM1
M+EA vs M-Down	Tyrosine metabolism	4	0.87	DCT, DDC, TH, AOX1

### Real-time PCR validation of DE genes which change the most in comparing group M vs C and M+EA vs M

**Fig 4:**
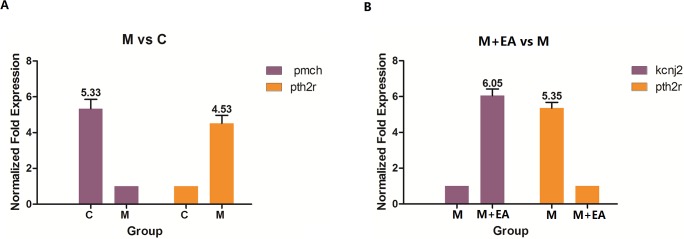
Real-time PCR analysis of genes *pmch*, *pth2r* and *kcnj2*. A: the expression of *pmch* and *pth2r* in group C and group M; B: the expression of *kcnj2* and *pth2r* in group M+EA and group M. The mRNA abundance was normalized using the housekeeping gene *β-actin*, and the relative expression levels were calculated using the 2^-ΔΔCt^ method. Three biological replicates were performed.

The expression levels of genes that changed the most in comparing groups M vs C and M+EA vs M were validated by Real-time PCR. In comparing group M vs C, genes *pth2r* (RefSeq: NM_031089.1) and *pmch* (RefSeq: NM_012625.1) were up-regulated and down-regulated for the most with the normalized expression level 6.643 and -6.64 respectively. In comparing group M+EA vs M, the expression level variation of genes *kcnj2* (RefSeq: NM_017296.1) and *pth2r* (RefSeq: NM_031089.1) were up-regulated and down-regulated the most significant and the normalized expression levels were 6.193 and -6.643. It is noteworthy that gene *pth2r* was up-regulated significantly in the model group compared with control group, but the expression of this gene back to normal after EA treatment. Description of *pth2r* showed that it encodes parathyroid hormone 2 receptor. Since the accumulation of parathyroid hormone is associated with aging, the regulation of parathyroid hormone maybe another mechanism of EA towards sub-acutely aging. The real-time PCR results reveals that expression trend of *pt*h2r (RefSeq: NM_031089.1), *pmch* (RefSeq: NM_012625.1) and *kcnj2* (RefSeq: NM_017296.1) were consistent with the cDNA microarray data, as can be seen in **Fig 4**.

## Discussion

Acupuncture is an effective alternative to drugs for the treatment of various kinds of disease by inserting thin needles deeply into the skin at specific points on the body. EA is a type of acupuncture wherein needles are attached to an apparatus that produces continuous electric pulses. As a traditional therapy, acupuncture has been used in China for thousands of years and these years began to gain increasing worldwide attention also. It is no doubt that acupuncture can improve or cure diseases [34]. However, the molecular mechanisms of how acupuncture effect have not been totally illustrated yet and need to be further studied.

According to previous report, acupuncture showed beneficial effect on anti-aging and age-related diseases [9]. As known, aging is an enormously complex phenomenon which involves immunosenescence, cognitive decline and so on [8]. In the field of brain, several genes have been proved to be related with aging. For example, the down-regulate of gene *Mdm-2*, a kind of apoptosis depressed gene can place a premium on apoptosis indirectly and causes neuron loss [35]. Besides, Kinesin family member 5C (Kif5C), expressed almost exclusively in murine brain, was also related with axonal elongation the maintenance of motor neurons [36, 37]. Our previous study demonstrate that EA at Guanyuan (CV 4), Zusanli (ST 36) and Baihui (DU 20) could delay aging by regulating T cells in proliferation, secretion of IL-2 and its receptor and improve the expression of CD8^+^,CD28^+^[26–27]. Moreover, content of IL-1β and IL-6 of serum and the expression of apoptosis genes can also regulated by EA [28]. According to the basic theory of traditional Chinese medicine, we should take the body as a whole in curing a disease and that is actually the essence of acupuncture therapy. Thus it is possible that acupuncture therapy may cure disease by bringing the expression change of a large sum of genes. As a result, in this study, the cDNA microarray analysis was explored to research the molecular mechanisms of EA in improving sub-acutely aging from the whole gene expression aspect.

D-galactose induced aging model was established in 1991 by a Chinese scholar named Gong Guoqing. This aging model can be obtained by D-galactose s.c. (D-Gal) intraperitoneal injection continuously, which is convenient, cheap and stable so that it has been used widespread [29]. The physiological changes of multiple organizations, multiple organ and multiple levels brought by this model were similar to those brought by natural aging. For example, the weight of D-Gal aging model rats decreased significantly (P<0.01) compared with the control group. The serum content of SOD decreased while that of MDA increased (P<0.05). Besides, the atrophy of thymus and the gain of weight of spleen were also brought by this model. The changing levels were similar to natural agingrats [29, 38–39]. In this study, in order to investigate the molecular mechanisms of sub-acutely aging and demonstrate the effect of electroacupuncture (EA) on the sub-acutely aging brain, the microarray analysis was explored to demonstrate differences between control group (C), the electroacupuncture group (M+EA) and the sub-acutely aging model group (M). In this study, the hippocampus area of ten rats in each group makes up one sample, and then the three samples from group C, group M and group M+EA were prepared for the RNA extraction. The pooling of samples has its pros and cons. It will lose the important information about between sample variability thus can not get an estimate of between sample variation. Moreover, if the pooled sample was failed, then all 10 samples were wasted. However, the pooling provides a more accurate estimate of the mean gene expression levels. In future researches, we may measure the gene expression levels for each of 10 samples and then use average gene expression level as the estimate of the expression level for this gene. In this way, we could not only get accurate estimate of gene expression level, but also get an estimate of between sample variation.

4052 DE genes were identified among three samples. As can be seen in **Fig 1**, the gene expression profile of the sub-acutely model group changed significantly compared with the control group, and the changes were brought by the effect of aging. Meanwhile, the gene expression also changed after the EA treatment, the gene expression profile of M+EA group was more similar to that of control group comparing with the model group. This result demonstrates that EA can bring the gene expression change in the hippocampus of sub-acutely rats in the whole aspect.

Subsequently, more analyses were carried out to classify DE genes and select important genes related with EA treatment. According to **Fig 2A**, comparing groups M vs C and M+EA vs M have 445 common DE genes. Further study reveals that among these 445 genes, 346 were down-regulated and 78 were up-regulated in group C and group M+EA compared with group M. This means EA treatment can reverse the expression change of 424 DE genes brought by the sub-acutely aging. Besides, 538 DE genes were specific to comparing group M+EA vs M, and these change were brought by the EA treatment (**Fig 3B and 3C**). The GO and KEGG pathway enrichment was then performed to category the DE genes in comparing groups M vs C and M+EA vs M. In comparing group M vs C, 5 KEGG pathways were up-regulated and 9 were down-regulated. While in comparing group M+EA vs M, 18 KEGG pathways were up-regulated and 5 were down-regulated. Further analysis reveals that EA treatment can reverse the change of five KEGG pathways caused by the sub-acutely aging, that are: phosphatidylinositol signaling system pathway, neuroactive ligand-receptor interaction, vascular smooth muscle contraction, melanogenesis and calcium signaling pathway. DE genes involved in these pathways were listed in **Table 5**. Changing of these pathways can be regarded as another mechanism of EA towards sub-acutely aging.

In order to validate the correction of microarray data, the gene expression levels of *pt*h2r (RefSeq: NM_031089.1), *pmch* (RefSeq: NM_012625.1) and *kcnj2* (RefSeq: NM_017296.1) were measured by real-time PCR. In comparing groups M vs C and M+EA vs M, these genes were up-regulated and down-regulated for the most. Noteworthy, *pth2r* was the most significant up-regulated gene in comparing group M vs C; it was also the most significant down-regulated gene in comparing group M+EA vs M, which means the expression of *pth2r* back to normal after EA treatment. Gene annotation showed that this gene is the parathyroid hormone 2 receptor encoding gene. Since the accumulation of parathyroid hormone is associated with aging, *pth2r* should be regarded as an important gene related with the sub-acutely aging. In group M+EA, the expression level of *pth2r* down regulated significantly to the level of that in group C, meaning that EA treatment has reverse the expression change of gene *pth2r*, which was another mechanism of EA against sub-acutely aging in rat’s brain.

To sum up, this study first compared and analyzed the differences of gene expression profile in the hippocampus of sub-acutely aging rats before and after the EA treatment. Important genes associated with sub-acutely aging and EA effects were identified, which contribute to our knowledge on the molecular mechanisms of EA towards sub-acutely aging and pave the way for the future study.

## Supporting information

S1 Table The comparison of gene expression profilebetween different samples. (XLS)

S2 Table Common elements in "M vs C-UP" and "M vs M+EA-UP"; Common elements in "M vs C-DOWN" and "M vs M+EA-DOWN". (XLSX)

S1 Fig The process of the experiment. (TIF)
