# Supplementary figures and images for: Electroacupuncture at Guanyuan (CV 4), Zusanli (ST 36) and Baihui (DU 20) regulate the aging-related changes in gene expression profile of the hippocampus in sub-acutely aging rats
Source: PLoS One. 2018 Jan 19;13(1):e0191623. doi: 10.1371/journal.pone.0191623 (PMC5774823; doi:10.1371/journal.pone.0191623)

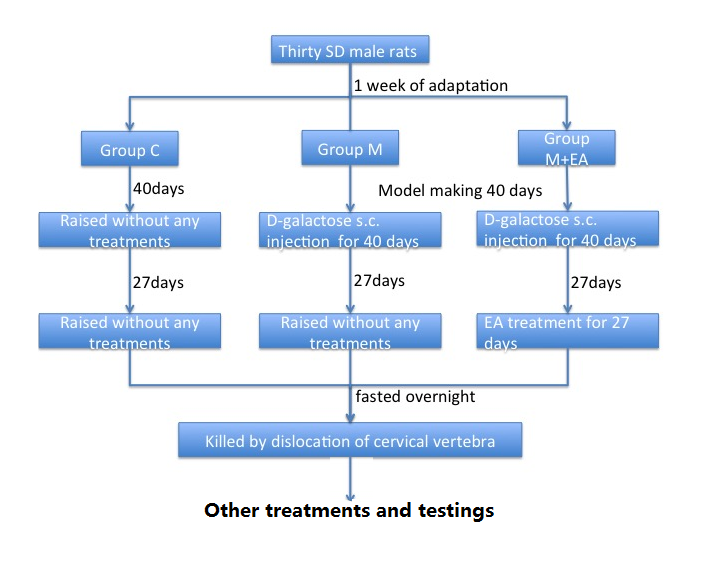

Supplement: S1 Fig — (TIF) [file pone.0191623.s003.tif]
